# Local brain oscillations and interregional connectivity differentially serve sensory and expectation effects on pain

**DOI:** 10.1126/sciadv.add7572

**Published:** 2023-04-19

**Authors:** Felix S. Bott, Moritz M. Nickel, Vanessa D. Hohn, Elisabeth S. May, Cristina Gil Ávila, Laura Tiemann, Joachim Gross, Markus Ploner

**Affiliations:** ^1^ Department of Neurology and TUM-Neuroimaging Center (TUM-NIC), TUM School of Medicine, Technical University of Munich (TUM), Munich, Germany.; ^2^ Institute for Biomagnetism and Biosignalanalysis, University of Münster, Münster, Germany.

## Abstract

Pain emerges from the integration of sensory information about threats and contextual information such as an individual’s expectations. However, how sensory and contextual effects on pain are served by the brain is not fully understood so far. To address this question, we applied brief painful stimuli to 40 healthy human participants and independently varied stimulus intensity and expectations. Concurrently, we recorded electroencephalography. We assessed local oscillatory brain activity and interregional functional connectivity in a network of six brain regions playing key roles in the processing of pain. We found that sensory information predominantly influenced local brain oscillations. In contrast, expectations exclusively influenced interregional connectivity. Specifically, expectations altered connectivity at alpha (8 to 12 hertz) frequencies from prefrontal to somatosensory cortex. Moreover, discrepancies between sensory information and expectations, i.e., prediction errors, influenced connectivity at gamma (60 to 100 hertz) frequencies. These findings reveal how fundamentally different brain mechanisms serve sensory and contextual effects on pain.

## Teaser

Sensory and expectation effects on pain are implemented by fundamentally different brain mechanisms.

## INTRODUCTION

Pain serves to protect the body. To this end, the brain translates sensory information about potential threat into an unpleasant experience and protective behavioral responses. However, this translation is shaped not only by sensory but also by contextual information, such as an individual’s expectations (*1*–*3*). Expectations can yield powerful and clinically relevant changes of the pain experience, for example, through placebo and nocebo effects (*4*–*7*). Moreover, contextual and expectation effects are particularly relevant for pathological aberrations of the pain experience in chronic pain disorders (*8*–*10*).

In the brain, pain is associated with complex patterns of neural activity in somatosensory, insular, cingulate, and prefrontal cortices as well as subcortical brain areas (*11*, *12*). Neurophysiological studies using electroencephalography (EEG), magnetoencephalography (MEG), and intracranial recordings have shown that this brain network yields complex temporal-spectral patterns of neural responses including evoked potentials and oscillatory responses at alpha (8 to 12 Hz), beta (13 to 30 Hz), and gamma (30 to 100 Hz) frequencies (*13*). In addition, it is increasingly recognized that not only the local brain activity but also the communication between brain regions, i.e., interregional brain connectivity, critically shapes the experience of pain (*14*–*20*).

Recent studies have started to unravel how these complex spatial-temporal-spectral patterns of brain activity serve sensory and contextual effects on pain. Functional magnetic resonance imaging (fMRI) studies have revealed that these effects are served by different spatial patterns of brain activity. For instance, patterns of brain activity termed the neurologic pain signature (NPS) and the stimulus intensity–independent pain signature (SIIPS) are particularly sensitive to sensory and contextual effects on pain, respectively (*21*, *22*). EEG studies have indicated that the temporal-spectral patterns of sensory and contextual effects on pain also differ (*23*–*25*). Specifically, evoked potentials and oscillatory responses to noxious stimuli are more sensitive to sensory information than to expectations (*23*–*25*). In contrast, the temporal-spectral pattern of expectation effects on pain has remained largely unclear so far. Mechanistic considerations suggest that contextual effects on pain such as expectations might be particularly shaped by interregional top-down connectivity between supra-modal and sensory brain regions at alpha and beta frequencies (*13*). Moreover, predictive coding (PC) frameworks of brain function (*26*, *27*) propose that discrepancies between sensory and expectation effects, i.e., prediction errors (PEs), are mediated by brain oscillations and connectivity at gamma frequencies (*28*–*30*). However, direct evidence for these hypotheses on how sensory and expectation effects on pain are implemented by local brain activity and interregional connectivity is lacking so far.

To better understand and directly compare how local brain oscillations and interregional connectivity serve sensory and contextual effects on pain, we reanalyzed data from an EEG experiment in which brief painful stimuli were applied to healthy human participants (*23*). Therein, sensory and contextual information was modulated by varying stimulus intensity and expectations about upcoming stimulus intensity, respectively. A previous analysis of the experiment focused on the functional significance of EEG responses that are commonly analyzed to assess the cerebral processing of pain (*23*). To this end, we analyzed evoked and oscillatory EEG responses in electrode space. We found clear evidence that these EEG responses are involved in signaling sensory information. By contrast, a Bayesian analysis provided evidence against an involvement of these responses in signaling expectations or PEs. It, thus, remained unclear, how the brain serves expectations and PEs in the processing of pain. Considering the complexity of expectation effects, we hypothesized that they are particularly served by interregional connectivity in brain networks associated with pain. Therefore, in the present study, we set out to assess and directly compare how local oscillatory brain activity and interregional connectivity in a core network of six brain regions associated with the processing of pain serve the effects of stimulus intensity and expectations on pain.

## RESULTS

**Fig. 1.**
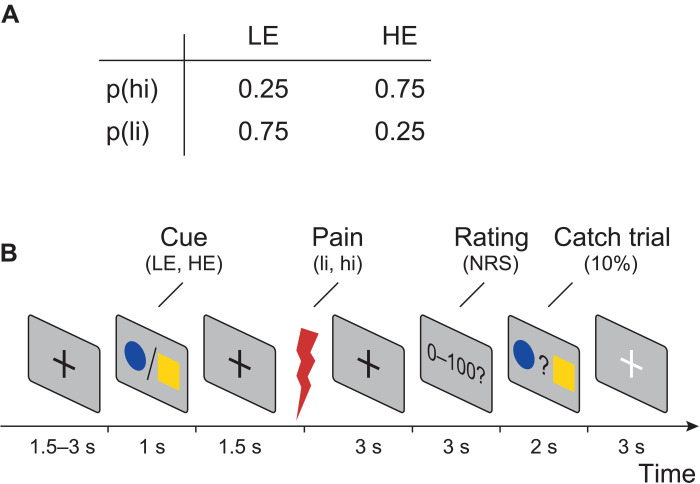
Experimental design. (**A**) Probabilities of different pain stimulus intensities [low intensity (li) and high intensity (hi)] for different levels of expectation [low expectation (LE) and high expectation (HE)]. (**B**) In each trial, a cue was presented that probabilistically predicted the intensity of a subsequent painful stimulus. Three seconds after the stimulus, a verbal pain rating was obtained from the participants. In 10% of the trials (catch trials), participants were visually prompted to indicate by a button press whether a HE or a LE cue had been presented to ensure that participants continuously paid attention to the visual cues. More details on the experimental design can be found in (*23*).

To investigate how the brain serves sensory and contextual influences on pain, we used a probabilistic cueing paradigm. We applied brief painful heat stimuli to the left hand and independently modulated stimulus intensity and expectations in a 2 × 2 factorial design. To modulate stimulus intensity, we applied painful stimuli of two different levels [high intensity (hi) and low intensity (li)]. To modulate expectations, the painful stimuli were preceded by one of the two visual cues, probabilistically indicating the intensity of the subsequent stimulus. The high expectation (HE) cue was followed by a hi stimulus in 75% of the trials and by a li stimulus in 25% of the trials. Conversely, the low expectation (LE) cue was followed by a hi stimulus in 25% of the trials and by a li stimulus in 75% of the trials. The experiment thus comprised four trial types (Fig. 1A): hi, HE (hiHE); hi, LE (hiLE); li, HE (liHE); li, LE, (liLE). In each trial, after the painful stimulus, the participants were asked to provide a rating of the perceived pain intensity on a numerical rating scale ranging from 0 (no pain) to 100 (maximum tolerable pain). Figure 1B shows the sequence of a single trial. The experiment included 160 trials per participant.

**Fig. 2.**
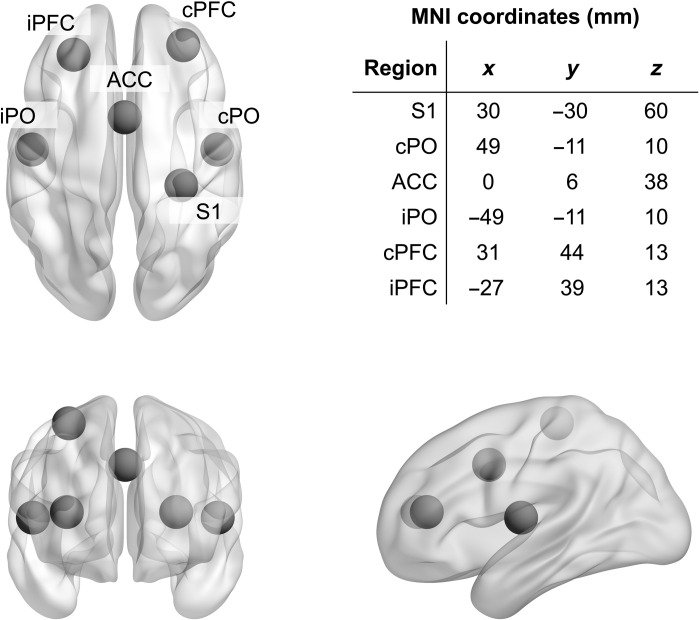
ROI and corresponding MNI coordinates. Axial, coronal, and sagittal view of the brain and the six regions of interest (ROIs). S1, contralateral primary somatosensory cortex; cPO and iPO, contra- and ipsilateral parietal operculum; ACC, anterior cingulate cortex; cPFC and iPFC, contra- and ipsilateral prefrontal cortex. Please note that the asymmetry of the PFC ROIs reflects the asymmetry of PFC sources found in (*31*).

We analyzed oscillatory brain activity and functional connectivity in a network of six brain regions (Fig. 2) known to play key roles in the cerebral processing of pain (*31*). The brain regions were the contralateral primary somatosensory cortex (S1), the contra- and ipsilateral parietal operculum (cPO and iPO, respectively; including the secondary somatosensory cortex and parts of the insular cortex), the anterior cingulate cortex (ACC), and the contra- and ipsilateral prefrontal cortex (cPFC and iPFC, respectively). Some of these brain regions are particularly associated with processing of sensory information (S1, cPO, and iPO), whereas others are more associated with supramodal cognitive and emotional processes (ACC, cPFC, and iPFC) (*11*, *12*). Coordinates for these six regions of interest (ROIs) were taken from human intracranial recordings that represent the gold standard for electrophysiological brain responses to pain stimuli (*31*). To assess oscillatory brain activity, we calculated frequency-specific power in source space. To assess functional connectivity between brain regions, we calculated the debiased weighted phase lag index (dwPLI) (*32*). Both local activity and interregional connectivity were assessed in the alpha (8 to 12 Hz), beta (14 to 30 Hz), and gamma (60 to 100 Hz) frequency bands. These frequency bands are known to exhibit changes in oscillatory power in response to brief painful stimuli (*33*–*36*) and play key roles in interregional communication in the brain (*37*). In addition, to assess the dominant direction of information flow in selected connections and frequency bands, we computed an asymmetry index on the basis of the partial directed coherence (PDC) measure (*38*) of directed functional connectivity.

**Fig. 3.**
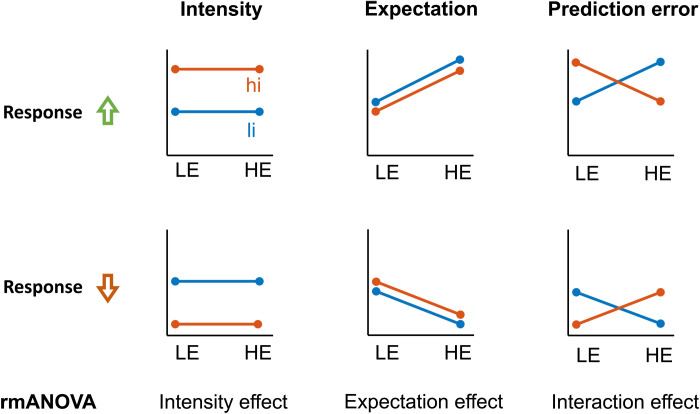
Possible response patterns indicating the effects of stimulus intensity, expectations, and (absolute) PEs. Effects of stimulus intensity (li and hi), expectations (LE and HE), and PEs were tested by means of repeated measures analyses of variance (rmANOVAs). An experimental modulation can lead to either a relative increase (first row) or relative decrease (second row) of oscillatory activity or connectivity.

To relate brain activity and connectivity to sensory and expectation effects on pain, we defined different patterns describing the relation between response variables and experimental manipulations (*39*, *40*). In particular, these patterns characterize how neural phenomena and pain ratings are linked to intensity, expectations, or discrepancies thereof (PEs) across the four trial types (Fig. 3). To formally link the data to these patterns, we performed repeated measures analyses of variance (rmANOVAs) (*41*) with the independent variables intensity and expectation. In these rmANOVAs, features signaling stimulus intensity and expectations would manifest as main effects, whereas features signaling PEs would manifest as interactions without distinguishing between absolute and aversive definitions of PEs. To allow for the interpretation of negative findings, we specifically performed Bayesian rmANOVAs (*41*). Results of frequentist analyses are provided in the Supplementary Materials.

### The effects of stimulus intensity and expectations on pain intensity ratings

**Fig. 4.**
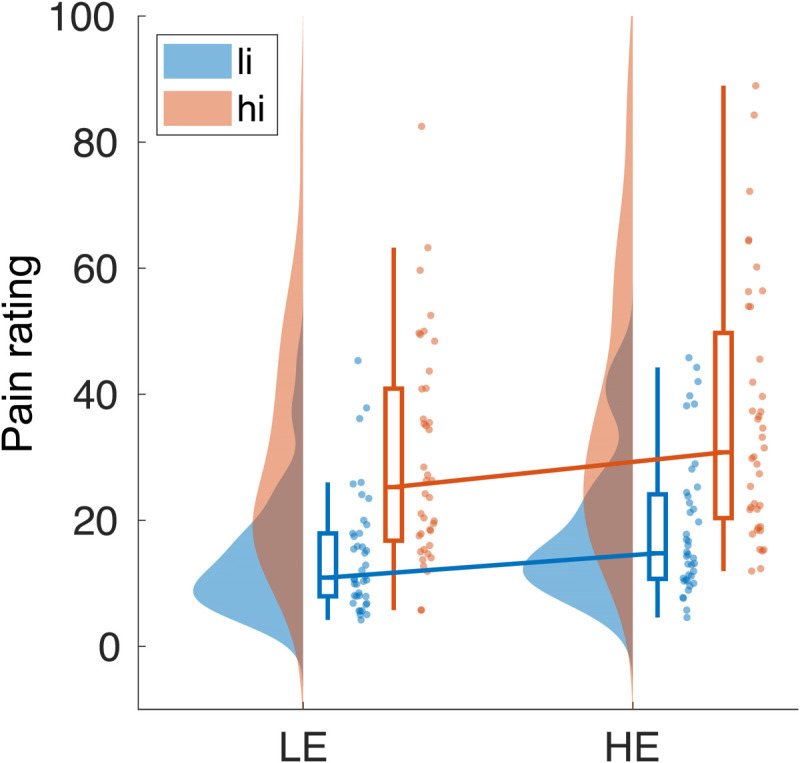
Effects of stimulus intensity, expectations, and PEs on pain ratings. Rain cloud plot (*67*) of pain ratings for two levels of stimulus intensity (li and hi) and expectation (LE and HE). A Bayesian rmANOVA yielded decisive evidence for main effects of stimulus intensity and expectation [Bayes factor (BF) = 1.1 × 10^21^ and BF = 5.5 × 10^2^, respectively]. Moreover, there was moderate evidence against an interaction (BF = 0.27).

Figure 4 shows pain intensity ratings for the four trial types. Analyses of pain ratings provided decisive evidence for main effects of intensity [Bayes factor (BF) = 1.1 × 10^21^] and expectation (BF = 5.5 × 10^2^) on pain ratings. Specifically, as expected, hi stimuli yielded higher pain ratings than li stimuli, and HE cues yielded higher pain ratings than LE cues. Moreover, there was moderate evidence against an interaction effect of intensity and expectation (BF = 0.27). Thus, the results confirmed that stimulus intensity and expectations shaped pain ratings.

### The effects of stimulus intensity and expectations on local oscillatory brain activity

**Fig. 5.**
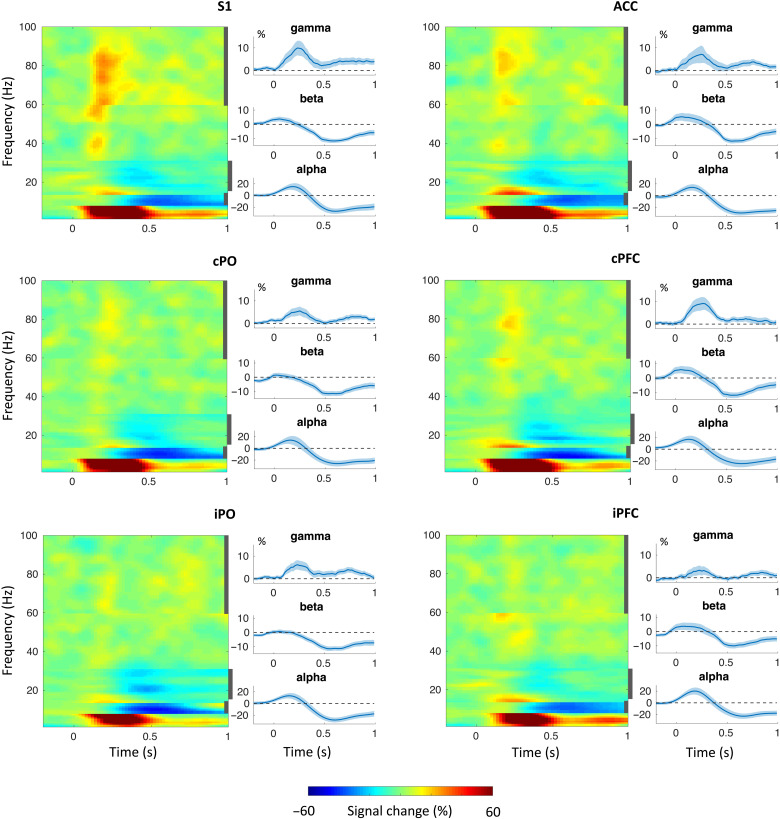
TFRs based on hi trials of local oscillatory brain activity in the six ROIs. The first and third columns show concatenated band specific time-frequency representations (TFRs) for all six ROIs. The sharp transitions in the TFRs are due to the employment of frequency band-specific spatial filters. The second and fourth columns show time courses of brain activity in the alpha, beta, and gamma band. Vertical, dark gray bars in the TFR plots indicate the frequency intervals based on which the time courses of brain activity were computed.

We first assessed how brief noxious stimuli influenced local oscillatory brain activity in the six ROIs. Time-frequency representations (TFRs; Fig. 5) indicated that noxious stimuli suppressed alpha and beta activity in all ROIs and increased gamma activity predominantly in S1. In addition, noxious stimuli yielded increases of activity at frequencies below 8 Hz, which reflect evoked potentials analyzed previously (*23*).

**Fig. 6.**
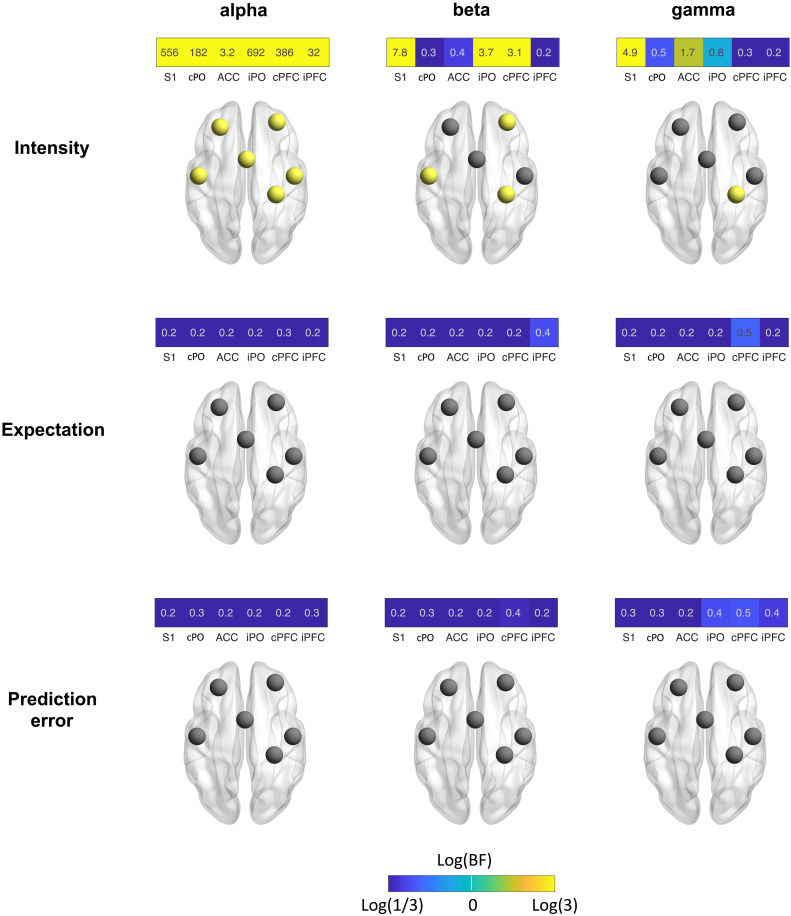
Effects of stimulus intensity, expectations, and PEs on local brain activity. Effects were assessed by Bayesian rmANOVAs with the factors intensity and expectation. The color of the tiles representing ROIs scales with the log of the BF. It ranges from blue (BF < 0.33, at least moderate evidence against an effect) to yellow (BF > 3, at least moderate evidence for an effect). Brain images display ROIs in yellow, which exhibit at least moderate evidence for an effect (BF > 3).

Next, we assessed how stimulus intensity and expectations influence local brain activity in our core network associated with pain processing. We therefore determined the power of brain activity in the six ROIs at alpha, beta, and gamma frequencies averaged across the 1-s poststimulus interval. The results of Bayesian rmANOVAs with the factors intensity and expectation are shown in Fig. 6. Figures S1 and S2 show corresponding results of frequentist statistics that are qualitatively similar to the results of Bayesian statistics.

We found that stimulus intensity modulated local brain activity at all frequency bands and in all ROIs. Strongest stimulus intensity effects were observed at alpha frequencies where we found moderate to decisive evidence for effects on oscillatory brain activity for all ROIs. In all ROIs, stronger stimuli yielded stronger suppressions of alpha activity (see fig. S3 for direction and strength of all effects). Weaker effects were observed at beta frequencies where we found moderate evidence for an intensity effect on brain activity in S1, iPO, and cPFC. In these ROIs, stronger stimuli yielded stronger suppressions of beta activity. In the gamma frequency band, we observed moderate evidence for an intensity effect on S1 brain activity with stronger stimuli inducing higher amplitudes of gamma activity. In contrast, we found weak to moderate evidence against effects of expectations or PEs on local brain activity at all frequency bands. Control analyses using shorter time windows showed qualitatively similar results (fig. S4). In summary, we found that stimulus intensity but not expectations or PEs influenced local oscillatory brain activity in response to brief painful stimuli.

### The effects of stimulus intensity and expectations on interregional functional connectivity

**Fig. 7.**
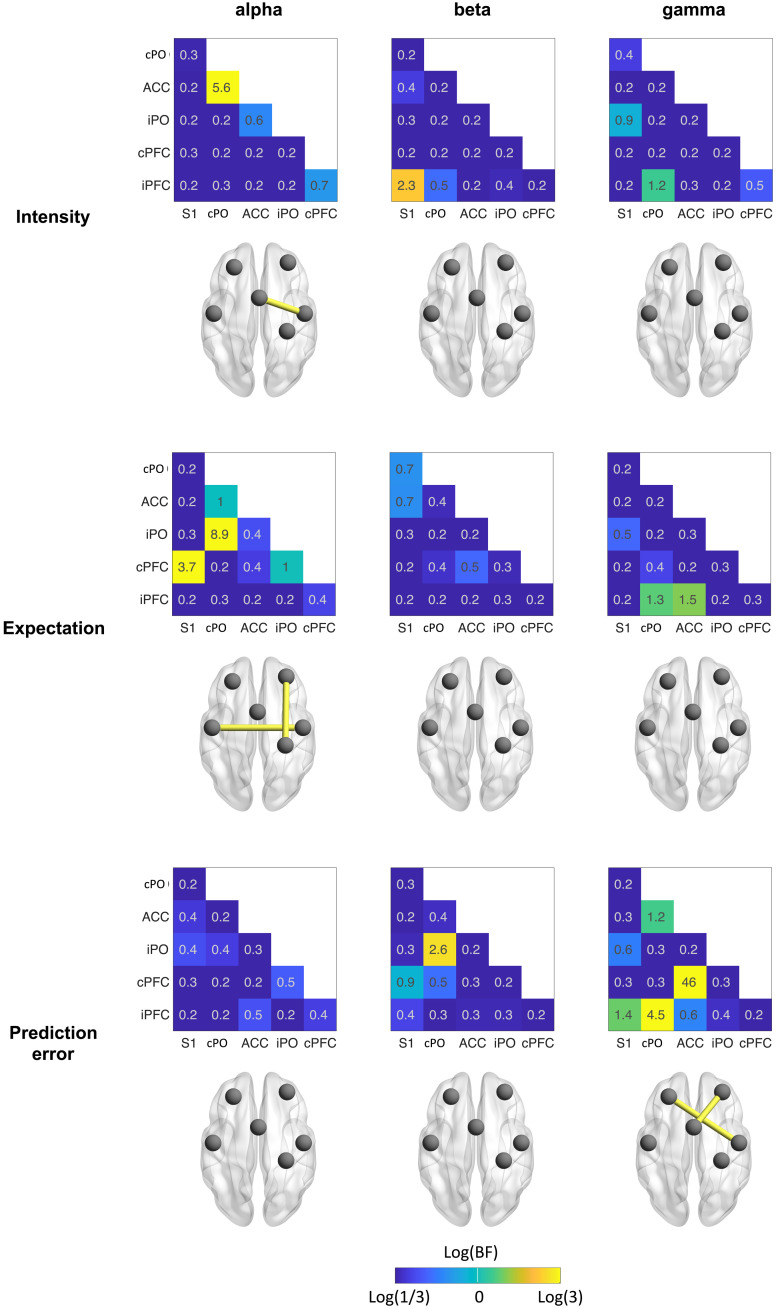
Effects of stimulus intensity, expectations, and PEs on interregional functional connectivity. Effects were assessed by Bayesian rmANOVA with the factors intensity and expectation. The color of the heat map tiles scales with the log of the BF. It ranges from blue (BF < 0.33, at least moderate evidence against an effect) to yellow (BF > 3, at least moderate evidence for an effect). Brain images display connections in yellow which exhibit at least moderate evidence for an effect (BF > 3).

We next investigated how stimulus intensity and expectations influenced communication in our core network associated with pain processing. We therefore determined pairwise interregional connectivity in a network of six ROIs resulting in 15 connectivity values. These analyses were performed separately for the alpha, beta, and gamma frequency bands in the 1-s poststimulus interval. Figure 7 shows the results of Bayesian rmANOVAs. Figures S1 and S2 show the corresponding results of frequentist statistics that are qualitatively similar to the results of Bayesian statistics.

We found moderate evidence for a stimulus intensity effect on the cPO-ACC connection in the alpha band. Here, connectivity was higher in the hi than the li condition. For most other connections and frequency bands, we found weak to moderate evidence against stimulus intensity effects.

Effects of expectation were found in the alpha band exclusively. We specifically observed moderate evidence for an expectation effect on the cPFC-S1 and iPO-cPO connections. In these connections, connectivity was lower in the HE than the LE conditions. For most other connections and frequency bands, we found weak to moderate evidence against expectation effects.

PE effects were observed in the gamma band exclusively. We found moderate to strong evidence for a PE effect on the cPFC-ACC and iPFC-PO connections. Specifically, the mean connectivity values of mismatch conditions (hiLE and liHE) were lower than those of nonmismatch conditions (liLE and hiHE). In other words, conditions involving a PE exhibited lower connectivity than those without a PE. For most other connections and frequencies, we observed weak to moderate evidence against a PE effect. Together, we found that stimulus intensity and expectation influenced connectivity at alpha frequencies, whereas PE effects were found at gamma frequencies.

### Direction of functional connectivity

**Fig. 8.**
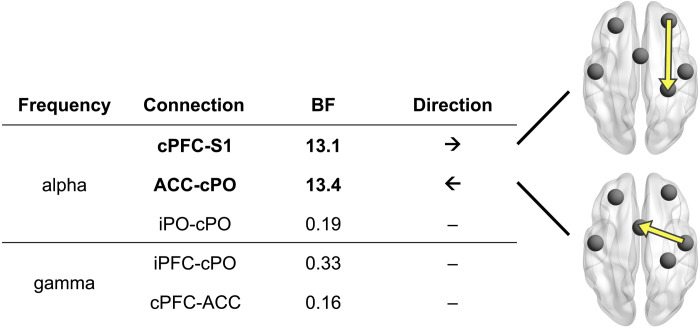
Direction of functional connectivity. Using an asymmetry score based on the PDC connectivity metric, we assessed the direction of information flow in connections that exhibited evidence for an effect in the previous connectivity analysis. Brain images depict connections with strong evidence for asymmetric information flow. The arrows indicate the dominant direction of information flow.

The previous analyses showed that stimulus intensity, expectations, and PEs modulated functional connectivity at alpha and gamma frequencies in a core network associated with pain processing. We were next interested to assess the direction of information flow for connections in which we found at least moderate evidence for intensity, expectation, and/or PE effects. To this end, we calculated an asymmetry score of directed connectivity between pairs of brain regions. The score was based on the bivariate PDC (*38*) measure and ranged from −1 to 1. The absolute value and the sign of the score indicate the strength and the direction of asymmetry, respectively. For the cPO-ACC connection, for which intensity effects were observed in the alpha band, we found strong evidence (BF = 13.4) that information flowed from cPO to ACC. For the cPFC-S1 connection, for which expectation effects were observed in the alpha band, we found strong evidence (BF = 13.1) that information flowed from cPFC to S1. For the other connections and frequency bands, we did not find evidence for an asymmetry of information flow. Thus, as summarized in Fig. 8, for connections showing intensity effects, we found information flow predominantly from sensory to higher-order brain areas. Conversely, for connections displaying expectation effects, we found information flow predominantly from higher-order to sensory brain areas. Figure S5 shows corresponding results of frequentist statistics that are qualitatively similar to the results of Bayesian statistics.

### Comparisons of stimulus intensity and expectations effects on local oscillatory brain activity and interregional functional connectivity

The previous analyses indicated that local brain activity and interregional connectivity differentially serve sensory and expectation effects on pain. We specifically observed that stimulus intensity shaped local brain activity more than interregional connectivity, while expectations and PEs shaped interregional connectivity more than local activity. To address this statistically, we conducted a Bayesian comparison of two types of models predicting the levels of stimulus intensity, expectation, and PE. The two types of models differed with respect to the variables they incorporate for their predictions. One type of model incorporated activity variables, and the other incorporated connectivity variables. We found decisive evidence that activity-type models predicted stimulus intensity better than connectivity-type models (BF_pow/conn_ > 10^5^). Conversely, there was decisive evidence that connectivity models predicted expectations (BF_conn/pow_ > 10^2^) and PEs (BF_conn/pow_ > 2 × 10^2^) better than activity models. Methodological details of the model comparison are provided in the Supplementary Materials.

### Summary

**Fig. 9.**
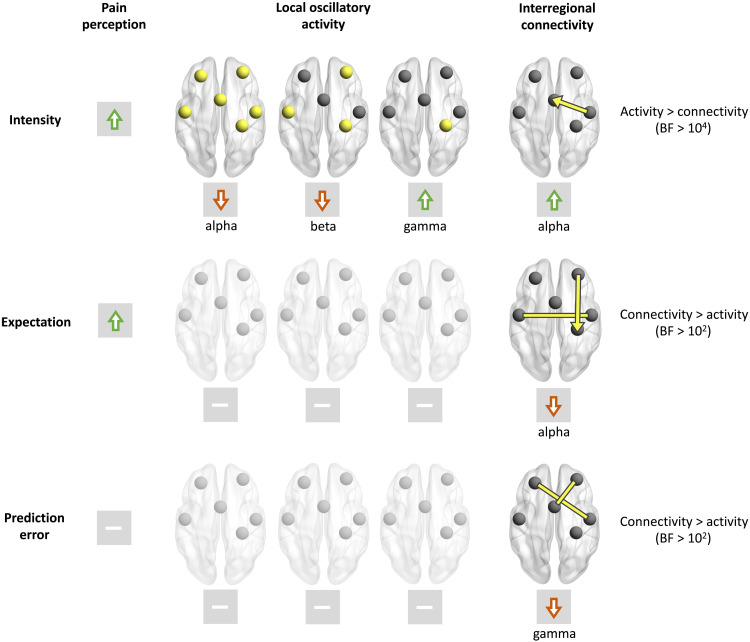
Synopsis of the effects of stimulus intensity, expectations, and PEs on pain perception, local brain activity, and interregional functional connectivity. Increases of stimulus intensity led to increases of pain ratings and local brain activity at gamma frequencies as well as to decreases of brain activity at alpha and beta frequencies. Expectations of stronger pain yielded increases of pain ratings and reduced connectivity between cPO and iPO and from cPFC to S1 at alpha frequencies. In contrast, expectations did not modulate local brain activity at any ROI and any frequency band. PEs did not change pain ratings or local brain activity but iPFC-cPO and cPFC-ACC connectivity at gamma frequencies. The last column shows the results of a Bayesian comparison of local brain activity and connectivity models predicting intensity, expectation, and PEs.

Figure 9 summarizes the main findings. On the behavioral level, both stimulus intensity and expectation modulated the perception of pain. As expected, both higher stimulus intensities and expectations of stronger stimuli evoked higher pain ratings. In the brain, stimulus intensity effects were predominantly associated with changes of local brain activity. Stronger stimuli yielded stronger responses to brief painful stimuli in alpha, beta, and gamma frequency bands. In contrast, expectation effects on pain were associated with changes of interregional functional connectivity but not with changes of local brain activity. We particularly found that expectation effects were associated with top-down connectivity at alpha frequencies from cPFC to S1 and with connectivity between cPO and iPO. PEs were associated with changes of gamma-band connectivity exclusively. Bayesian model comparisons confirmed the differential involvement of local activity and interregional connectivity in sensory and expectation effects on pain. Specifically, stimulus intensity has a stronger influence on local brain activity than on interregional connectivity. Vice versa, expectations and PEs shape interregional connectivity more than local brain activity.

## DISCUSSION

In the present study, we investigated how the brain serves sensory and contextual effects on pain. To this end, we applied noxious stimuli to healthy human participants and independently modulated stimulus intensity and expectations. Pain ratings confirmed that stimulus intensity and expectation both influenced pain perception. Analyses of EEG recordings revealed that sensory and expectation effects on pain were served by fundamentally different brain mechanisms. In a core network associated with the processing of pain, sensory information shaped local oscillatory brain activity rather than interregional functional connectivity. In contrast, expectation and PEs influenced interregional functional connectivity but not local oscillatory brain activity.

### Sensory and expectation effects on local oscillatory brain activity and interregional functional connectivity

We observed that sensory information shapes local oscillatory brain activity more than interregional connectivity. The effects of stimulus intensity on local oscillatory activity in various frequency bands are in accordance with previous EEG and MEG studies (*24*, *33*, *42*, *43*). However, the effects of stimulus intensity on local brain activity and interregional connectivity have not been directly compared so far.

We further observed that expectations influenced interregional functional connectivity but not local oscillatory brain activity. To the best of our knowledge, expectation effects on functional connectivity have not yet been investigated by neurophysiological recordings. A few studies have investigated expectation effects on local oscillatory brain activity (*24*, *25*, *44*, *45*). Their findings were inconsistent. Some studies found that expectations of high pain were associated with increased alpha activity (*24*, *44*), and others report unchanged (*25*) or decreased alpha activity (*45*). The present findings do not rule out any expectation effects on local brain activity. However, the crucial finding here is not the lack of expectation effects on local oscillatory activity, but that expectation effects on connectivity are stronger than on local oscillatory activity. Expectation effects were not observed at all but only few connections at certain frequencies and locations indicating the functional, spectral, and spatial specificity of expectation effects on connectivity. Furthermore, the aggregate model comparisons that integrate all connections at all frequencies provide direct evidence that interregional connectivity is more involved in signaling expectations than local activity.

### Expectation and PE signaling in the processing of pain

We found that expectation and PEs influenced connectivity at alpha/beta and gamma frequencies, respectively. This observation can be interpreted with reference to PC frameworks of brain function. PC is a general theory used to explain how perception arises from the integration of sensory information and expectations (*26*). The framework proposes that the brain maintains an internal model of the environment that continuously generates predictions about sensory input. Discrepancies between these predictions and the actual sensory evidence, i.e., PEs, serve to adjust the internal model. In this way, the brain allocates its limited resources to events that are behaviorally relevant and useful for updating predictions, i.e., learning. It has been suggested that alpha and beta oscillations serve the signaling of predictions, whereas gamma oscillations have been proposed to signal PEs (*28*–*30*, *39*). The present findings are in good accordance with this framework. They specify that expectation effects on pain might be particularly related to connectivity at alpha frequencies from the prefrontal to the somatosensory cortex. Specifically, expecting less pain was associated with relatively stronger connectivity. This implies that alpha band connectivity might be mechanistically involved in an active down-regulation of nociceptive input. PEs, on the other hand, were reflected in reduced gamma connectivity, indicating that they are signaled in the brain in terms of a disruption of interregional communication that is in line with a recent study on PE signaling in the processing of pain (*24*). These effects might reflect absolute (unsigned) or aversive (negative) PEs as both would manifest as interactions in the rmANOVAs. However, they do not reflect signed PEs as those would manifest as combinations of stimulus intensity and expectation effects that we have not observed for any activity or connectivity feature.

### Distinct brain mechanisms serve sensory and expectation effects on pain

The key finding of our study is that sensory and expectation effects on pain are served by distinct brain mechanisms. Previous fMRI studies have already revealed that sensory and contextual effects on pain are associated with different spatial patterns of brain activity. For instance, one spatial pattern of brain activity termed the NPS is much more sensitive to sensory than to contextual effects on pain (*46*, *47*). Vice versa, another pattern of brain activity termed the SIIPS is sensitive to contextual but not to sensory effects on pain (*22*). Moreover, a spatial dissociation between the encoding of sensory information and expectations has also been found within the insular cortex (*39*).

Our results extend these findings by showing that not only the spatial brain activity patterns serving sensory and contextual effects on pain differ but also these effects are served by fundamentally different neurophysiological mechanisms. Sensory effects predominantly occurred in local brain oscillations, whereas expectation effects were exclusively observed in interregional connectivity. The dissociation of sensory and expectation effects suggests that both physiological phenomena are partially independent of each other.

These findings might have implications for the understanding, assessment, and treatment of clinical pain conditions. In acute pain, which is predominantly shaped by sensory information, assessing and modulating local oscillatory brain activity might be appropriate. In contrast, in chronic pain, which is often largely detached from sensory information, interregional connectivity might be more informative than local activity. Such a close association between interregional connectivity and chronic pain is in accordance with studies using fMRI (*15*, *48*, *49*) and recent EEG studies on connectivity in chronic pain (*50*, *51*) and psychiatric disorders (*52*). In this way, the present findings can help to guide the development of biomarkers of acute and chronic pain. Beyond, our results might inform the search for neuronal targets for invasive and noninvasive interventions aiming at alleviating pain.

### Limitations

When interpreting our findings, certain limitations should be considered. First, in our paradigm, the effects of expectations on pain perception were weaker than the effects of stimulus intensity. The lack of expectation effects on local brain activity might therefore reflect the weak expectation effects on pain perception, and other paradigms with stronger expectation effects on perception might well modulate local brain activity. However, the central finding of the present study is not the absolute strength of sensory and expectation effects but that the patterns of sensory and expectation effects on local brain oscillations and brain connectivity fundamentally differ. The strength of perceptual effects might well determine the strength of neurophysiological effects but is unlikely to fundamentally change the difference in the patterns of sensory and expectation effects on brain activity and connectivity. We are therefore confident that the present findings reflect a fundamental difference in the brain mechanisms serving sensory and expectation effects on pain.

Second, to modulate pain, we manipulated participants’ expectations. Expectations are a particularly powerful and clinically highly relevant modulator of pain (*4*–*7*). However, it is unclear whether the present observations are specific to expectation-induced modulations of pain or whether they generalize to other cognitive and contextual modulations of pain.

Third, we applied brief experimental pain stimuli to healthy human participants. It is unclear whether these findings can be translated to other experimental and clinical types of pain. It remains to be investigated whether the findings generalize to chronic pain conditions in which other brain mechanisms apply and in which the brain undergoes substantial structural and functional plasticity (*11*, *53*).

Fourth, we did not use individual head models for EEG source reconstruction. Thus, more subtle activity and/or connectivity effects might not have been detected.

### Summary

Together, the present study shows that sensory and expectation effects on pain are served by distinct brain mechanisms. Sensory effects on pain are served by changes of local oscillatory brain activity, whereas expectation effects and discrepancies between sensory information and expectations are served by changes of interregional functional connectivity. These results provide basic science insights into the brain mechanisms of pain and analgesia. They specifically advance the understanding of how the brain serves key modulations of the subjective experience of pain. Beyond, they can inform the development of novel tools for the assessment and treatment of clinical pain conditions.

## MATERIALS AND METHODS

### Participants

The study was performed in healthy human participants at the University Hospital of the Technical University of Munich (TUM). Written informed consent was obtained from all participants before the experiment. The Ethics Committee of the Medical Faculty of the TUM approved the study protocol. The study was preregistered at ClinicalTrials.gov (NCT04296968) and conducted in accordance with the latest version of the Declaration of Helsinki. It followed recent guidelines for the analysis and sharing of EEG data (*54*). Inclusion criteria were right-handedness and age > 18 years. Exclusion criteria were pregnancy, neurological or psychiatric diseases, and regular intake of medication (aside from contraception and thyroidal medication). Severe internal diseases (e.g., diabetes) and skin diseases (e.g., psoriasis and vitiligo), previous surgeries at the head or spine, current or recurrent pain, metal or electronic implants, and any previous side effects associated with thermal stimulation constituted additional exclusion criteria.

For the current rmANOVA design (one group, four measurements), an assessment of statistical power using G*Power (*55*) yielded a sample size estimate of 36 participants with a power of 0.95, an alpha level of 0.05, and medium effect sizes of *f* = 0.25 [corresponding to an η^2^ of 0.06; (*56*)].

The original study recruited 58 healthy human participants [29 females; age, 24.0 ± 4.3 years (means ± SD)]. Ten participants were excluded due to either the absence of pain or low pain ratings [<10 on a numerical rating scale from 0 (no pain) to 100 (maximum tolerable pain)] during the familiarization run (*n* = 8), excessive startle responses in response to painful stimulation during the training run (*n* = 1), or technical issues with the response box used during catch trials (*n* = 1). To ensure robust estimates of connectivity values, we here additionally excluded participants with less than 10 trials remaining after the raw data cleaning procedure described below (*n* = 8). The final dataset used here thus comprised 40 participants (all right-handed; 21 females; age, 23.4 ± 2.9 years). Average anxiety and depression scores were below clinically relevant cutoff scores of 8 of 21 (*57*) on the Hospital Anxiety and Depression Scale (anxiety, 3.2 ± 2.2; depression, 0.9 ± 1.2) (*58*).

### Procedure

The objective of this analysis was to assess how sensory and contextual modulations are served by local brain activity and interregional brain connectivity. The experiment involved two levels of noxious stimulus intensities (hi and li) and two types of visual cues (HE and LE), resulting in four experimental conditions. The visual cues probabilistically predicted the intensity of the subsequent noxious stimulus. The HE cue was followed by a hi stimulus in 75% of the trials and by a li stimulus in 25% of the trials. Vice versa, the LE cue was followed by a hi stimulus in 25% of the trials and by a li stimulus in 75% of the trials (Fig. 1A).

Figure 1B depicts the sequence of events for each trial. After a variable fixation period ranging from 1.5 to 3 s, a visual cue (either blue dot or yellow square) was displayed for 1 s. A brief painful heat stimulus was applied 1.5 s after cue offset. Three seconds after the painful stimulus, participants were visually prompted to provide a verbal rating of the perceived pain intensity on a numerical rating scale ranging from 0 (no pain) to 100 (maximum tolerable pain in the context of the experiment). To ensure that participants continuously paid attention to the visual cues, participants were visually prompted to indicate by a button press whether a HE or a LE cue had been presented in 10% of the trials (catch trials). An average accuracy of 95.6 ± 0.1% indicated that participants successfully focused on the task during the entire experiment. Trials were separated by a 3-s period during which a white fixation cross was presented.

The experiment consisted of four runs with 40 trials each [hiHE (*n* = 15), hiLE (*n* = 5), liLE (*n* = 15), and liHE (n = 5)], resulting in total trial numbers of hiHE (*n* = 60), hiLE (*n* = 20), liLE (*n* = 60), and liHE (*n* = 20). Runs were separated by short breaks of ∼3 min. Pairings of visual cues with stimulus intensities were balanced across participants.

Before the experiment, the participants were familiarized with the stimulation and the intensity rating procedure by applying a sequence of 10 heat stimuli. Next, participants were informed about the pairing between cues and stimulus intensities, and a training run comprising 16 trials was conducted. This was to ascertain that all participants were aware of the pairing and to minimize learning during the main experiment. During the experiment, participants sat in a comfortable chair. They wore protective goggles and listened to white noise on headphones to eliminate effects of ambient sounds. Please see (*23*) for additional details.

### Stimulation

A laser pulse with a wavelength of 1340 nm, a duration of 4 ms, and spot diameter of approximately 7 mm was used to apply painful stimuli to the left hand (*59*). For li and hi stimuli, the stimulus intensity was set to 3 and 3.5 J, respectively. These stimulus intensities are known to consistently elicit painful sensations of discriminable intensity (*59*). The stimulation site was slightly changed after each stimulus to avoid tissue damage and habituation or sensitization.

### Recordings and preprocessing

Brain activity was recorded using actiCAP snap/slim with 64 active sensors (Easycap) placed according to the extended 10-20 system and BrainAmp MR plus amplifiers (Brain Products, Munich, Germany). During the recording, sensors were referenced to FCz and grounded at Fpz. The signals were sampled at 1000 Hz (0.1-μV resolution) and band-pass–filtered between 0.016 and 250 Hz, while impedances were kept below 20 kilohms.

Figure S6 summarizes the preprocessing and analysis steps. The BrainVision Analyzer software (version 2.1.1.327, Brain Products, Munich, Germany) was used for preprocessing. First, raw signals were low-pass–filtered with a cutoff frequency of 225 Hz. After down-sampling to a rate of 500 Hz, a 1-Hz high-pass filter (fourth-order Butterworth) and a band-stop filter between 49 and 51 Hz filter removing line noise were applied. An independent component (IC) analysis based on the extended infomax algorithm was then conducted on the basis of the −4.2- to 3.2-s peri-stimulus time windows of the EEG data. Subsequently, ICs representing artifacts originating from eye movements or muscles were removed from the unfiltered EEG data (*60*) using visual inspection. Moreover, data segments of 400 ms centered around data samples with amplitudes exceeding ±100 μV and data jumps exceeding 30 μV were automatically marked for rejection. Last, the data were inspected visually, and the remaining artifacts were manually marked for rejection. All signals were re-referenced to the average reference. The cleaned data were exported to MATLAB (version R2019b, MathWorks, Natick, MA), and further analyses were performed using FieldTrip [version 20210411; (*61*)]. Data were segmented into epochs ranging from −4 to 3 s in peri-stimulus time, and all trials with marked artifacts or pain ratings of zero were excluded. This resulted in 49.5 ± 8.5, 16.8 ± 2.8, 18.0 ± 1.6, and 52.9 ± 4.2 trials per participant in the liLE, liHE, hiLE, and hiHE conditions, respectively. To assure that all analyses for the different trial types were eventually performed on the same number of trials, we matched the numbers of trials. Figure S7 shows details of the trial matching procedure.

### Source model

To project sensor-level time series to source level, we used linearly constrained minimum variance (LCMV) beamformers (*62*) implemented in FieldTrip (*61*). Frequency-specific array-gain LCMV spatial filters for alpha, beta, and gamma frequencies were constructed on the basis of a lead field and a frequency-specific covariance matrix. A boundary element approximation of a realistically shaped, three-shell head model was used as the lead field. For each individual and frequency band, the covariance matrix was computed from the band-pass–filtered, −1- to 1-s (peri-stimulus time) concatenated data segments of all (nonrejected) trials. To ensure a robust computation of the inverse of the covariance matrix, we used Tikhonov regularization as implemented in FieldTrip with a regularization parameter value of 5% of the average sensor power. The fixed orientation of the lead field for every source location was chosen to maximize the spatial filter output. Source-level signals were then obtained by applying the frequency-specific LCMV operator to the corresponding band-pass–filtered sensor-level time series.

### Assessment of source-level TFRs

Source-level TFRs were obtained using the following procedure: First, we projected the band-pass–filtered sensor-level signals to source space using five frequency-specific LCMV spatial filters (i.e., for frequencies < 8 Hz, 8 to 12 Hz, 13 to 30 Hz, 30 to 60 Hz, and 60 to 100 Hz). For each ROI, we generated TFRs as well as time courses of alpha, beta, and gamma brain activity. The TFRs are based on Hanning-tapered data. Time courses of brain activity were computed on the basis of moving time windows and using a Slepian multitaper approach (see below). TFRs and time courses of brain activity were computed from data segments with widths of 500 and 250 ms for frequencies below and above 30 Hz, respectively. Both TFRs and time courses of brain activity are displayed as percentage change relative to a baseline period ranging from 0.75 to 0.25 s before the stimulus. To maximize the signal-to-noise ratio for visualization, the results represent the grand average across participants and hi trials.

### Analysis of local brain activity

Local oscillatory brain activity was assessed as frequency-specific source power of the six ROIs. First, source-level time series band-pass–filtered to the frequency band of interest were obtained using the beamformer described above. For these signals, we computed the power of the frequency in the middle of the frequency band of interest using a Slepian multitaper approach (*63*). The spectral smoothing width was set to one half of the width of the frequency band of interest. In this way, the power value incorporates information of the entire frequency band of interest. We computed source power in the alpha (8 to 12 Hz), beta (14 to 30 Hz), and gamma (60 to 100 Hz) frequency bands for each trial. We then averaged power values across trials for each condition and subject. To allow for the comparison of the effects on local brain activity to those on brain connectivity, the analysis was primarily performed on a 1-s poststimulus interval. However, sensor-level findings indicate that the effects of painful stimuli on oscillatory brain activity are usually confined to shorter time windows. Specifically, pain-induced suppressions of brain activity at alpha and beta frequencies occur at latencies between 500 and 900 ms and between 300 and 600 ms, respectively (*33*, *42*). In addition, pain-induced increases of brain activity at gamma frequencies occur between 150 and 350 ms (*43*). We therefore performed control analyses using these shorter time intervals (see fig. S2 for results).

### Analysis of interregional connectivity

Connectivity analyses were performed on the 1-s poststimulus intervals of the source-level time series of the six ROIs. First, we computed the source-level cross-spectral density of each participant using a multitaper approach analogous to the one used for the computation of source power.

To assess functional connectivity, we calculated the dwPLI (*32*) on the basis of all trials of each condition and for every subject. We selected the dwPLI measure due to its insensitivity to volume conduction effects.

For the assessment of the direction of connectivity, we used an asymmetry score on the basis of bivariate PDC (*38*). Specifically, for two ROIs A and B, the bivariate PDC analysis yields two values, PDC_A➔B_ and PDC_B➔A_, representing the directed connectivity strength from A to B and from B to A, respectively. We cast these two values into a single asymmetry score (PDC_A➔B_ − PDC_B➔A_)/(PDC_A➔B_ + PDC_B➔A_), ranging from −1 to 1. A large absolute value of the asymmetry score indicates a strong asymmetry of directed connectivity. The sign of the asymmetry score reveals the predominant direction of information flow. Direction of connectivity was calculated for connections that had shown intensity, expectation, and/or PE effects in previous analyses. For connections with evidence for an intensity or expectation effect in the Bayesian ANOVA, we included all trial types in the computation of the asymmetry score. For connections with evidence for an interaction effect, we included trials with a mismatch between cue and intensity only.

### Statistical analyses

For each of the four trial types (liLE, hiLE, liHE, and hiHE), behavioral and EEG measures were computed on the basis of an identical number of trials. This number was determined as the minimum number of available trials across the four trial types. Details of the trial matching procedure can be found in the Supplementary Materials (fig. S7).

Building upon previous investigations (*39*, *40*), we made specific predictions about how EEG responses signaling stimulus intensity, expectations, PEs, or combinations thereof are modulated across the four trial types. To formally test these predictions, we performed rmANOVAs with the independent variables stimulus intensity and expectation. In these rmANOVAs, responses signaling stimulus intensity and expectations would manifest as main effects, whereas responses signaling PEs would manifest as interactions. This applies to definitions of PEs as absolute (unsigned) PE and to aversive PE, i.e., a PE occurs only if the stimulus is more painful than expected. To quantify effects and to facilitate interpretation of negative findings, we performed Bayesian rmANOVAs (*41*). In Bayesian rmANOVAs, the BF is the ratio between the likelihood of the data given the effect of interest and the likelihood of the data without the effect of interest. BF > 3 and BF > 10 indicate moderate and strong evidence in favor of the effect of interest, whereas BF < 0.33 and BF < 0.1 indicate moderate and strong evidence against the effect of interest, respectively (*41*). We considered a neural measure or pain rating as corresponding to the intensity or expectation pattern if there was at least moderate evidence for the corresponding main effect. Accordingly, we considered a neural measure or pain rating as corresponding to the PE pattern if the evidence for an interaction effect of intensity and expectation was at least moderate.

Last, for the assessment of asymmetry of information flow, we tested asymmetry scores against 0 using a nonparametric Bayesian *t* test. All parametric Bayesian analyses were conducted using the BayesFactor package in R (*64*); for nonparametric Bayesian *t* tests, we used freely available R code (*65*).

### Bayesian model comparison

We intended to statistically assess whether an experimental contrast (intensity, expectation, or PE) is associated more strongly with local activity or interregional connectivity. To this end, we conducted a Bayesian comparison of power-based and connectivity-based models predicting the levels of intensity, expectation, and PE. Specifically, we computed the Bayesian evidence of logistic models mapping individual power and connectivity values to the probability of observing a certain level of intensity, expectation, or PE. In the analysis, we consider *N*_pow_ = 6 power values and *N*_conn_ = 15 connectivity values in each of the *N*_freq_ = 3 frequency bands. For each of the three types of experimental contrasts, this resulted in *N*_freq_ × *N*_pow_ = 18 model evidence values for the power-based models and *N*_freq_ × *N*_conn_ = 45 model evidence values for the connectivity-based models. The BF for, e.g., the intensity manipulation reported in the manuscript, is the average of the 18 power-based model evidence values divided by the average of the 45 connectivity-based model evidence values. For the factor expectation and the interaction between expectation and intensity, i.e., PE, we proceeded analogously. The derivation of Bayesian model comparisons for logistic regression models follows the description in (*66*) and is provided in the Supplementary Materials.

## Notes

View/request a protocol for this paper from *Bio-protocol*.
