# Supplementary material for: Local brain oscillations and interregional connectivity differentially serve sensory and expectation effects on pain
Source: Sci Adv. 2023 Apr 19;9(16):eadd7572. doi: 10.1126/sciadv.add7572 (PMC10115421; doi:10.1126/sciadv.add7572)
Supplement: Supplementary file 1 — Figs. S1 to S8 Supplementary Methods [file sciadv.add7572_sm.pdf]

Science Advances
AAAS

# Supplementary Materials for

## Local brain oscillations and interregional connectivity differentially serve sensory and expectation effects on pain

Felix S. Bott *et al.*

Corresponding author: Markus Ploner, markus.ploner@tum.de

*Sci. Adv.* **9**, eadd7572 (2023)
DOI: 10.1126/sciadv.add7572

**This PDF file includes:**

Figs. S1 to S8
Supplementary Methods

# Supplementary Materials

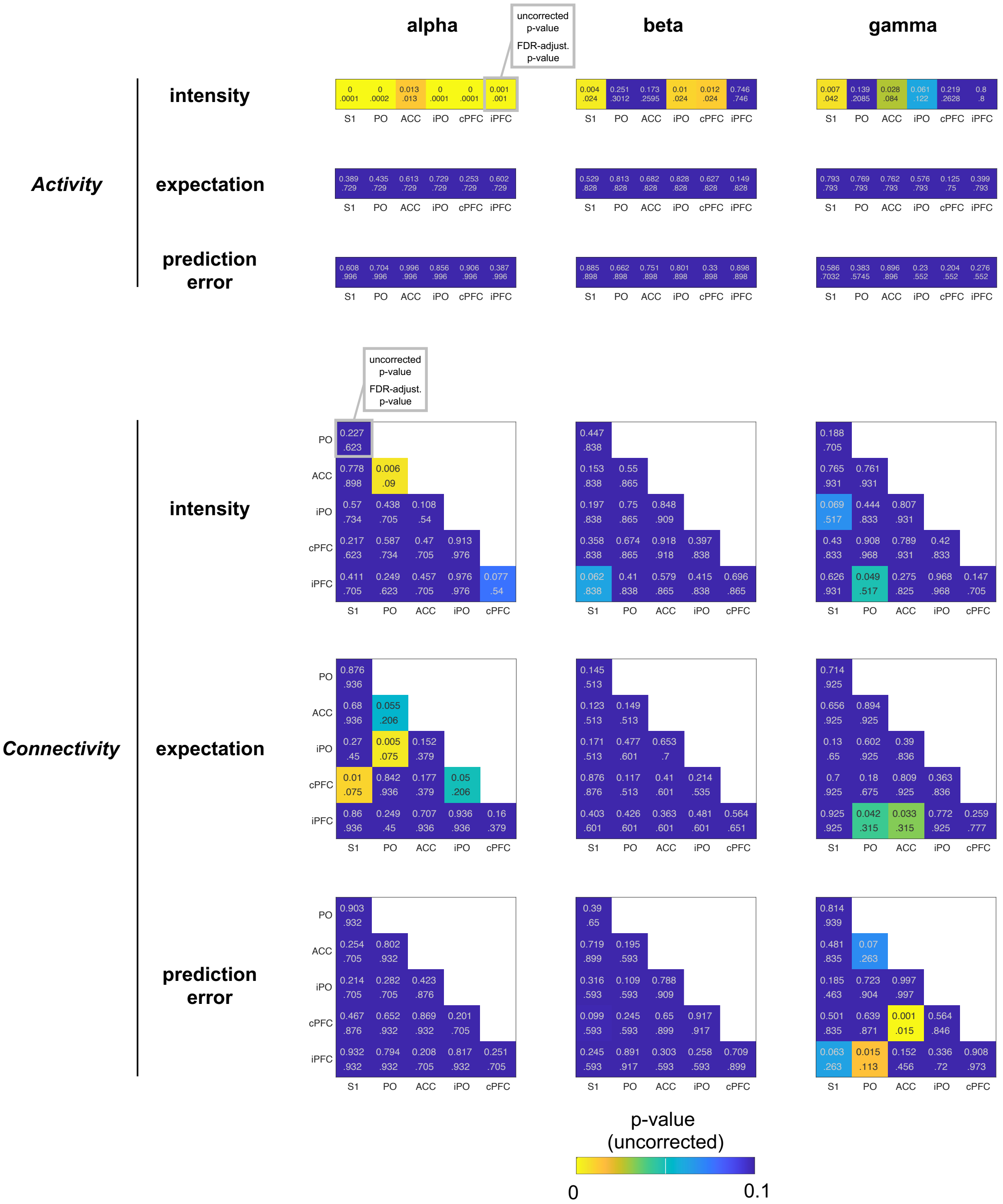


**Figure S1. Frequentist analysis of the effects of stimulus intensity, expectations, and prediction errors on local brain activity (top three panel rows) and inter-regional functional connectivity (bottom three panel rows).** Effects were assessed by a frequentist rmANOVA with factors intensity and expectation. The top number in each tile shows the uncorrected p-value, the bottom number the FDR-adjusted p-value. The adjustment was performed across all 15 connections and 6 ROIs in the case of inter-regional connectivity and local activity, respectively. The color of the tiles scales with the uncorrected p-value. Corresponding F-values and $\eta^2$-value are shown in Figure S2.

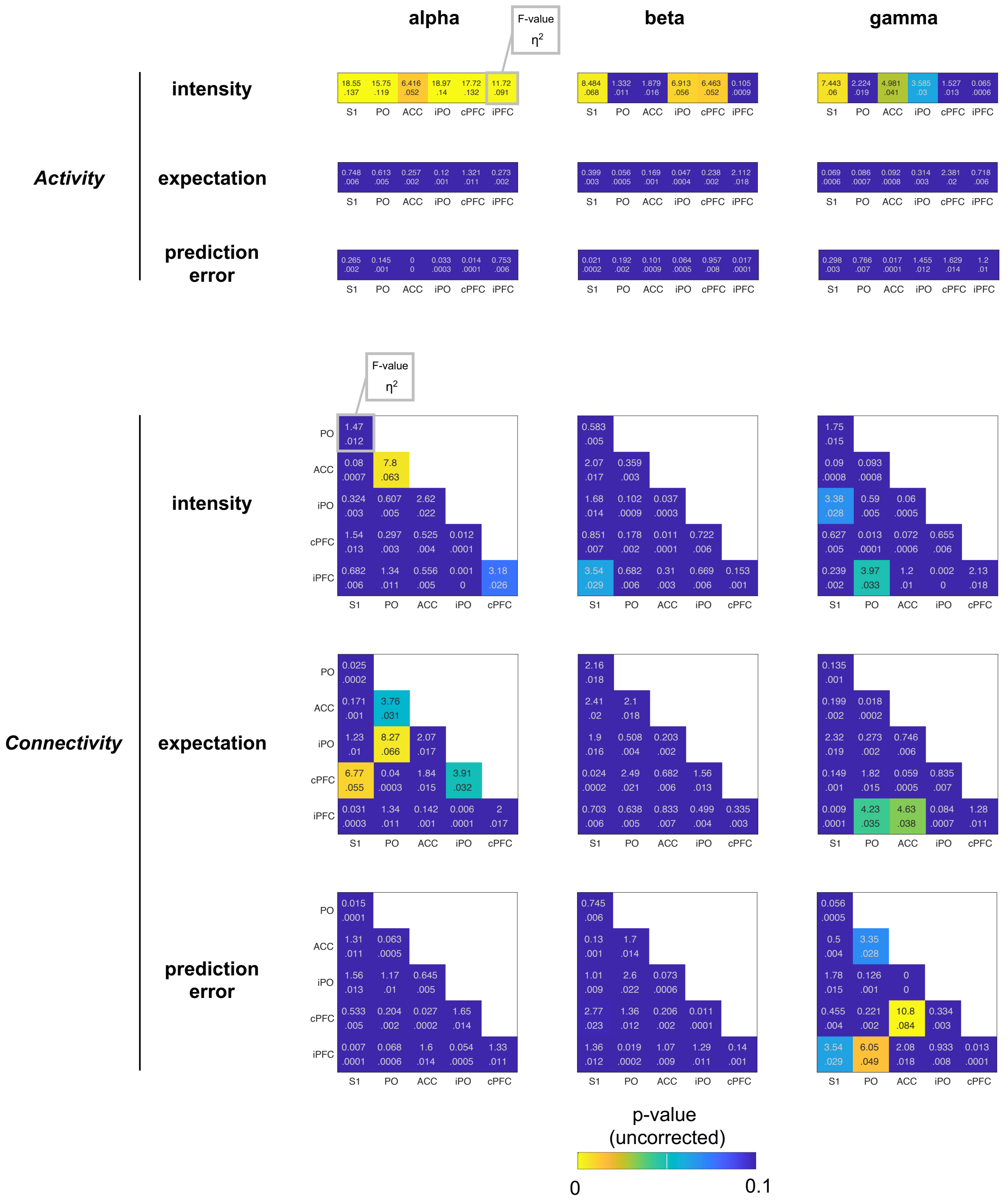


**Figure S2. Frequentist analysis of the effects of stimulus intensity, expectations, and prediction errors on local brain activity (top three panel rows) and inter-regional functional connectivity (bottom three panel rows).** Effects were assessed by a frequentist rmANOVA with factors intensity and expectation. The top number in each tile corresponds to the F-value, the bottom number is the $\eta^2$-value. Corresponding uncorrected and FDR-adjusted p-values are shown in Figure S1. The color of the tiles scales with the uncorrected p-value.

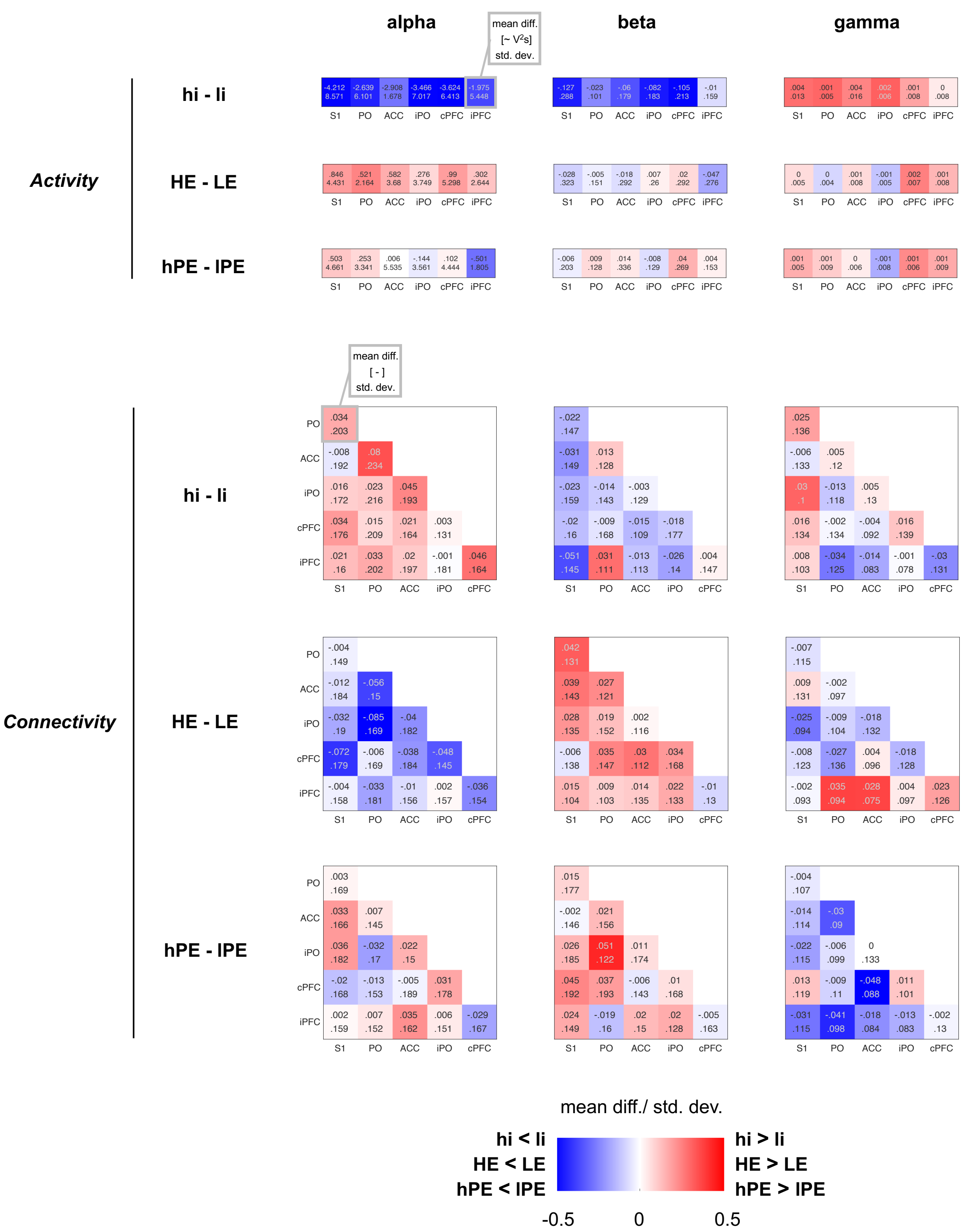


**Figure S3. Direction and strength of intensity, expectation, and prediction error effects on local brain activity (top three panel rows, [$V^2$s]) and inter-regional functional connectivity (bottom three panel rows [ - ]).** The figure shows average mean differences between the two levels of intensity, expectation, and prediction error. The top number in each tile indicates the mean difference between two types of conditions, the bottom number indicates the standard deviation of these mean differences across participants. The tile color scales with the quotient of the mean difference and standard deviation, thus indicating both the direction of a condition difference (blue and red indicating smaller and larger values in the first relative to the second condition, respectively) as well the as the magnitude of the difference relative to its variability across subjects (more intense colors indicating a larger difference). For the intensity contrast (hi - li), the mean difference is defined as the mean across all participants of the difference between the averaged hi-conditions

(mean[HEhi, LEhi]) and averaged li-conditions (mean[HEli, LEli]). Accordingly, for the expectation contrasts (HE - LE), the mean difference is the mean across all participants of mean[HEhi, HEli] - mean[LEhi, LEli]. For the prediction error contrast (hPE - IPE), the mean difference is the mean across all participants of mean[HEli, LEhi] - mean[HEhi, LEli].

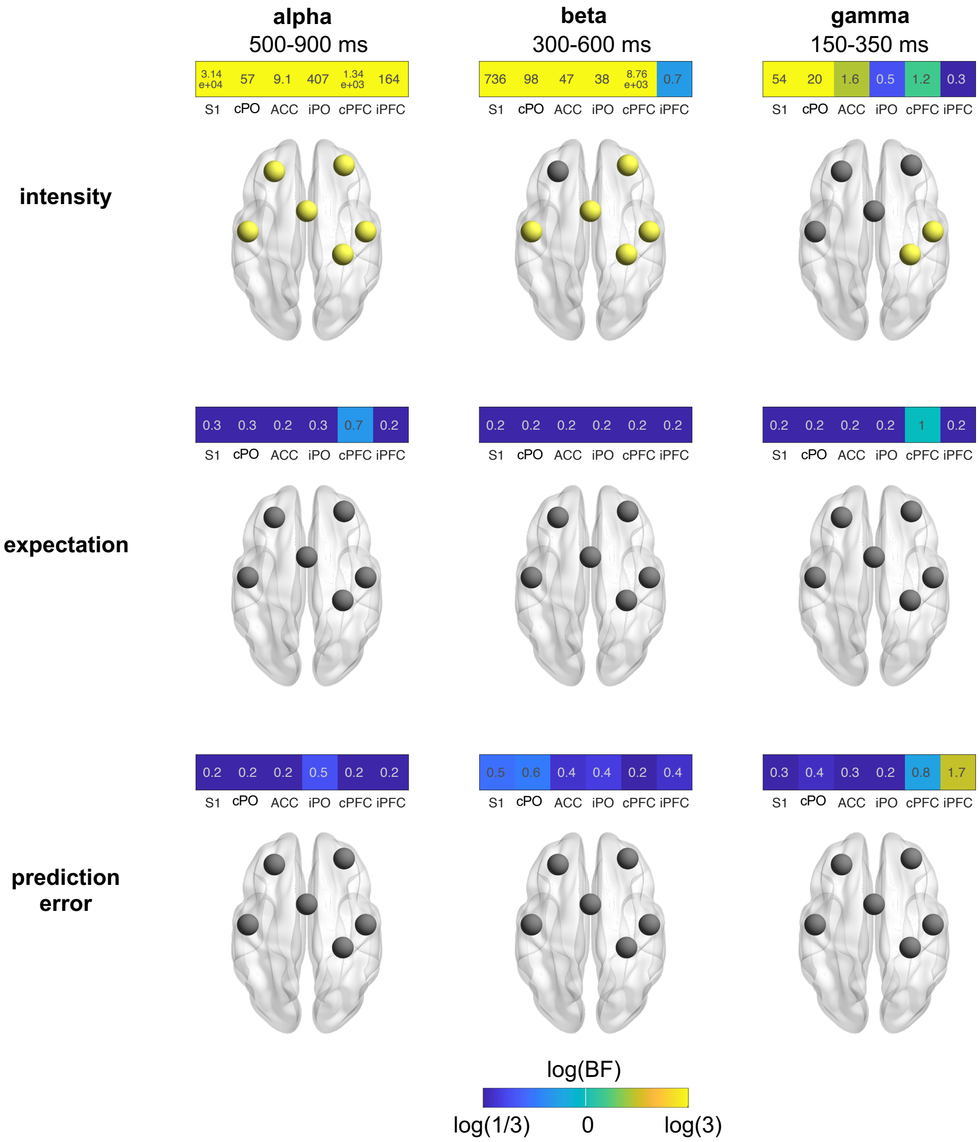


**Figure S4. Control analysis for the effects of stimulus intensity, expectations, and prediction errors on local brain activity.** Power at alpha, beta, and gamma frequencies was quantified using the time windows 500-900 ms, 300-600 ms, and 150-350 ms, respectively. Heat maps indicate Bayes factors of a Bayesian rmANOVA with factors intensity and expectation. The color of the heat map tiles scales with the log of the Bayes factor. It ranges from blue (BF < 1/3, at least moderate evidence against an effect) to yellow (BF > 3, at least moderate evidence for an effect). Brain schematics display ROIs in yellow which exhibit at least moderate evidence for an effect (BF > 3).

| Frequency | Connection | p-value | Direction |
|---|---|---|---|
| alpha | **cPFC – S1** | **0.0050** | → |
| | **ACC – cPO** | **0.0033** | ← |
| | iPO – cPO | 0.531 | - |
| gamma | iPFC – cPO | 0.243 | - |
| | cPFC – ACC | 0.906 | - |

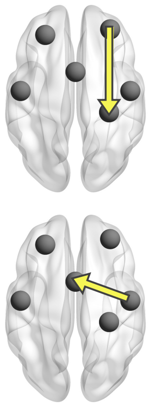

**Figure S5. Frequentist analysis of the direction of functional connectivity.** Using an asymmetry score based on the PDC connectivity metric, we assessed the direction of information flow in connections which exhibited evidence for an effect in the previous connectivity analysis. P-values are results from frequentist t-tests of PDC asymmetry. This figure is the frequentist counterpart to Figure 8 in the results section of the main text.

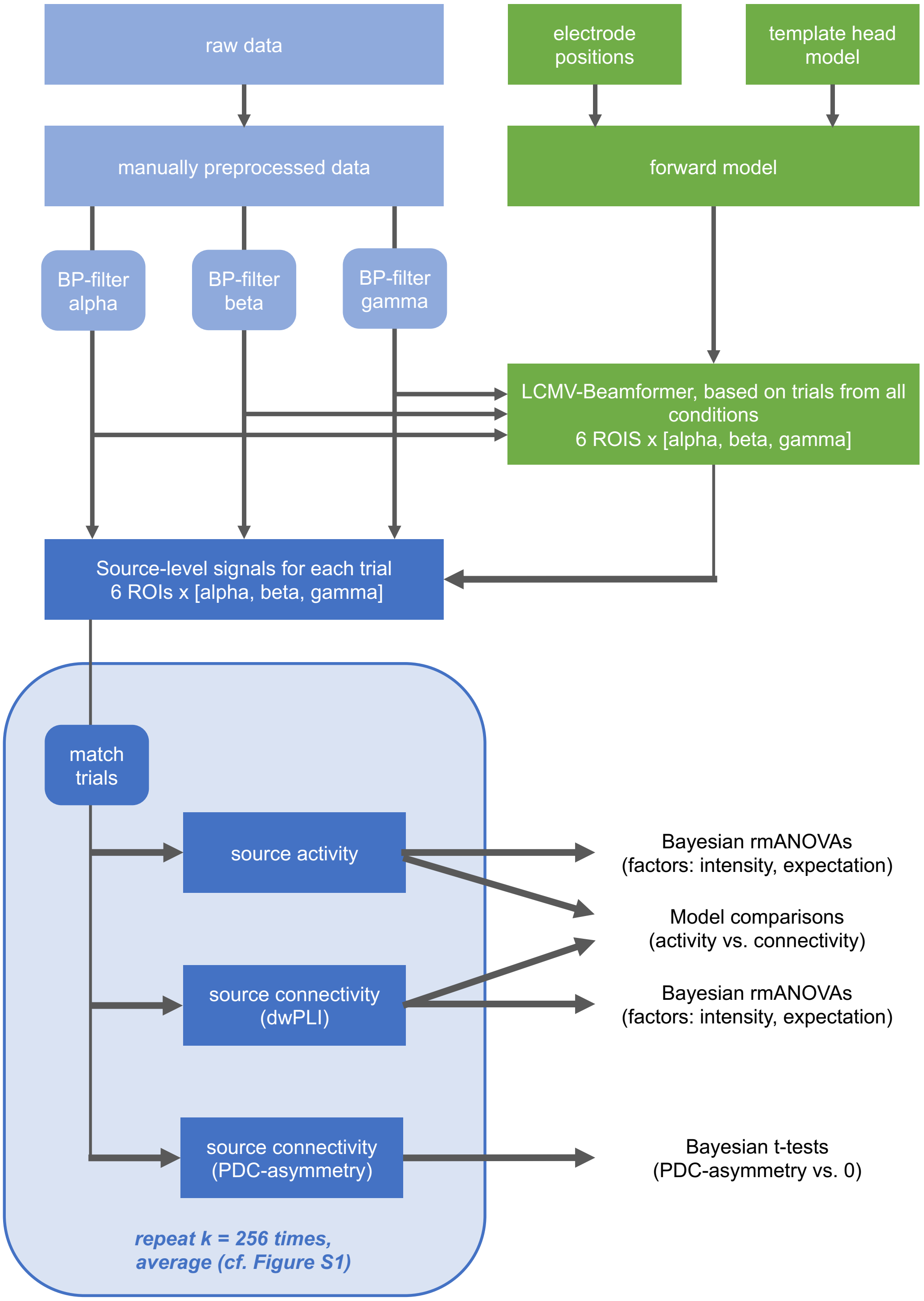


**Figure S6. Outline of the analysis pipeline.**

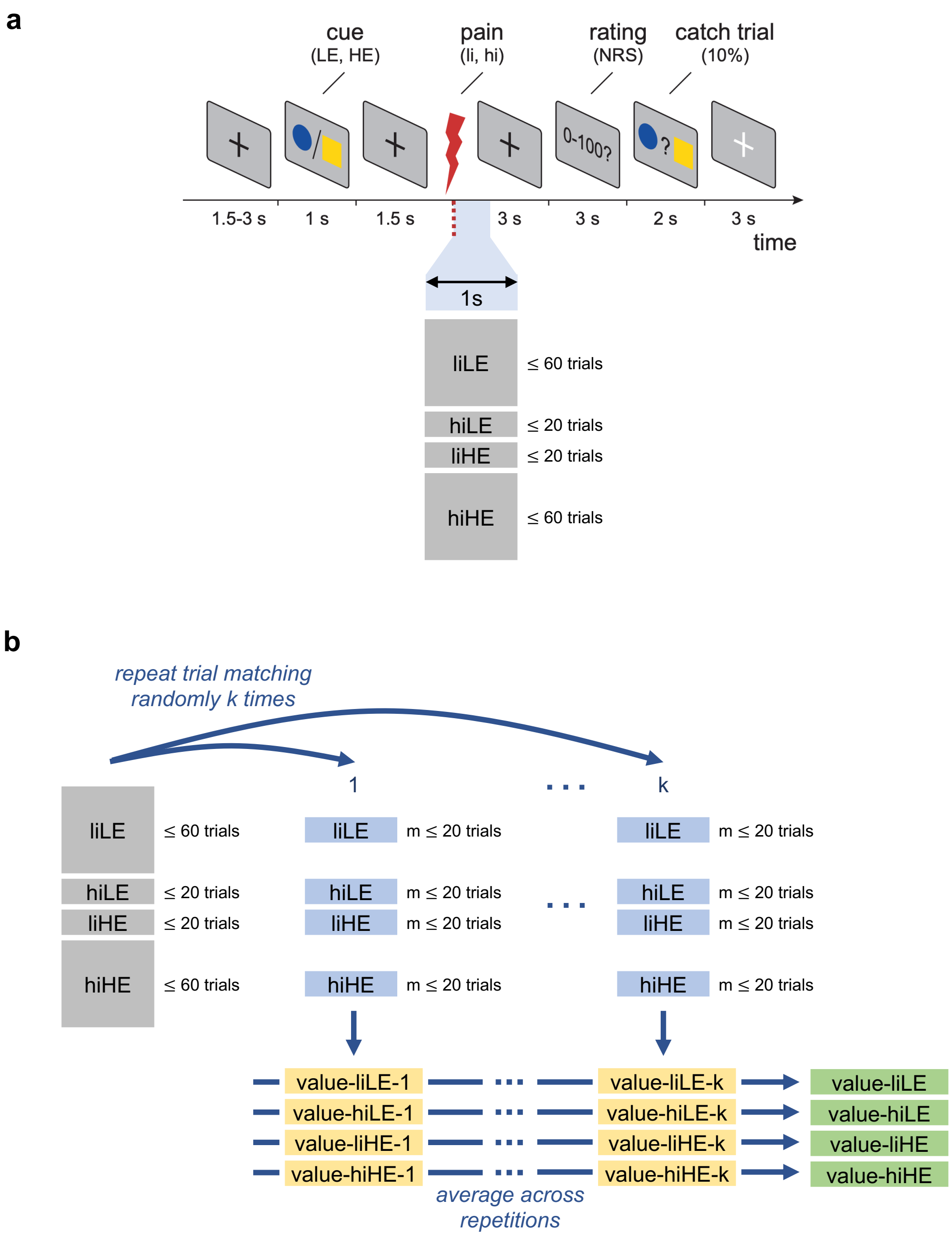


**Figure S7.** Trial matching. (a) The experiment comprised 160 trials per participant. In each trial, a cue (LE/HE) was presented which probabilistically predicted the intensity of a subsequent painful stimulus (li/hi). A LE(HE) cue preceded a li(hi) stimulus in 75% and a hi(li) stimulus in 25% of trials. This design in combination with the rejection of bad trials resulted in an unbalanced number of trials across the four trial types liLE, hiLE, liHE, and hiHE. (b) In order to circumvent a sample-size bias problem, all neural measures were computed based on the same number ($m \leq 20$) of trials. The matching of trial sets was done randomly and repeated $k = 256$ times. For each trial set, the corresponding values (pain rating/power/connectivity) were computed. This resulted in k estimates per measure which were averaged to obtain a single value per measure.

**Supplementary Methods: Bayesian model comparison**

The objective is to statistically assess whether an experimental contrast (intensity, expectation, or PE) is associated more strongly with regional activity or inter-regional connectivity. To this end, we conducted a Bayesian comparison of power-based and connectivity-based models predicting the levels of intensity, expectation, and PE. The derivations in this section follow the description in (67).

Say there are $N$ participants to be included in the analysis. Let $\mathbf{S}^i, \mathbf{E}^i, \mathbf{P}^i$, and $\mathbf{C}^i$ be the data vectors associated with participant $i \in 1, \ldots, N$. The vectors $\mathbf{S}^i = [0,0,1,1]^\mathsf{T}$ and $\mathbf{E}^i \in [0,1,0,1]^\mathsf{T}$ encode the levels of stimulus intensity (li: 0, hi: 1) and expectation (LE: 0, HE: 1), respectively. The vectors $\mathbf{P}^i = \left[P^i_{\text{LEli}}, P^i_{\text{HEli}}, P^i_{\text{LEhi}}, P^i_{\text{HEhi}}\right]^\mathsf{T}$ and $\mathbf{C}^i = \left[C^i_{\text{LEli}}, C^i_{\text{HEli}}, C^i_{\text{LEhi}}, C^i_{\text{HEhi}}\right]^\mathsf{T}$ contain the corresponding values of power and connectivity, respectively. In the following, we will solely focus on the comparison of models discriminating low and high intensity. The derivation of the comparison of models for the expectation and PE contrasts is analogous.

First, to arrive at a single binary dependent and a single continuous independent variable (per model), we average the data across the two levels of expectation. In addition, to account for the repeated measures design of the experiment, we center the participant-level independent variable at 0. Thus, formally:

$$
\begin{aligned}
\bar{\mathbf{S}}^i &= \begin{bmatrix} 0 \\ 1 \end{bmatrix} \\
\bar{\mathbf{P}}^i &= \begin{bmatrix} \left(P^i_{\text{LEli}} + P^i_{\text{HEli}} - P^i_{\text{LEhi}} - P^i_{\text{HEhi}}\right)/4 \\ \left(P^i_{\text{LEhi}} + P^i_{\text{HEhi}} - P^i_{\text{LEli}} - P^i_{\text{HEli}}\right)/4 \end{bmatrix} \\
\bar{\mathbf{C}}^i &= \begin{bmatrix} \left(C^i_{\text{LEli}} + C^i_{\text{HEli}} - C^i_{\text{LEhi}} - C^i_{\text{HEhi}}\right)/4 \\ \left(C^i_{\text{LEhi}} + C^i_{\text{HEhi}} - C^i_{\text{LEli}} - C^i_{\text{HEli}}\right)/4 \end{bmatrix}
\end{aligned}
$$

The data of all participants is combined in vectors $\bar{\mathbf{S}} = [\bar{\mathbf{S}}^{1\mathsf{T}}, \ldots, \bar{\mathbf{S}}^{N\mathsf{T}}]^\mathsf{T}$, $\bar{\mathbf{P}} = [\bar{\mathbf{P}}^{1\mathsf{T}}, \ldots, \bar{\mathbf{P}}^{N\mathsf{T}}]^\mathsf{T}$, and $\bar{\mathbf{C}} = [\bar{\mathbf{C}}^{1\mathsf{T}}, \ldots, \bar{\mathbf{C}}^{N\mathsf{T}}]^\mathsf{T}$. To be able to use the same prior for all independent variables, the data vectors of the independent variables are scaled by their standard deviation:

$$
\begin{aligned}
\hat{\mathbf{S}} &= \bar{\mathbf{S}}/\text{std}(\bar{\mathbf{S}}) \\
\hat{\mathbf{P}} &= \bar{\mathbf{P}}/\text{std}(\bar{\mathbf{P}}) \\
\hat{\mathbf{C}} &= \bar{\mathbf{C}}/\text{std}(\bar{\mathbf{C}})
\end{aligned}
$$

In both models to be compared, the probability of observing the high intensity (hi) level is modeled as a logistic function:

$$
p(hi|x; \mu, \sigma) = \frac{1}{1 + \exp\left((x - \mu)/\sigma\right)} \,,
$$

where $\mu$ and $\sigma$ are parameters controlling the location and scale of the logistic function, respectively. Depending on the type of model, the continuous independent variable $x$ represents either a power or a connectivity value. The likelihood of the data given the model parameters thus is

$$p(\hat{\mathbf{S}}, \mathbf{x}|\mu, \sigma) = \prod_{k=1}^{2N} p\,(hi|x_k; \mu, \sigma)^{\hat{S}_k} \left(1 - p(hi|x_k; \mu, \sigma)\right)^{(1-\hat{S}_k)}.$$

with

$$\mathbf{x} = \begin{cases} \hat{\mathbf{P}} & \text{power model (pow)} \\ \hat{\mathbf{C}} & \text{connectivity model (conn).} \end{cases}$$

For the computation of the Bayesian model evidence a prior distribution over the model parameters $\mu$ and $\sigma$ must be specified. Here, we select a bivariate standard normal distribution:

$$\begin{bmatrix} \mu \\ \sigma \end{bmatrix} \sim \mathcal{N}\left(\begin{bmatrix} \mu \\ \sigma \end{bmatrix};\ \mathbf{0}, \mathbf{I}\right),$$

where $\mathbf{0}$ and $\mathbf{I}$ are the zero vector and identity matrix, respectively. Figure S3 shows graphs of logistic functions for several parameter values drawn from the prior distribution.

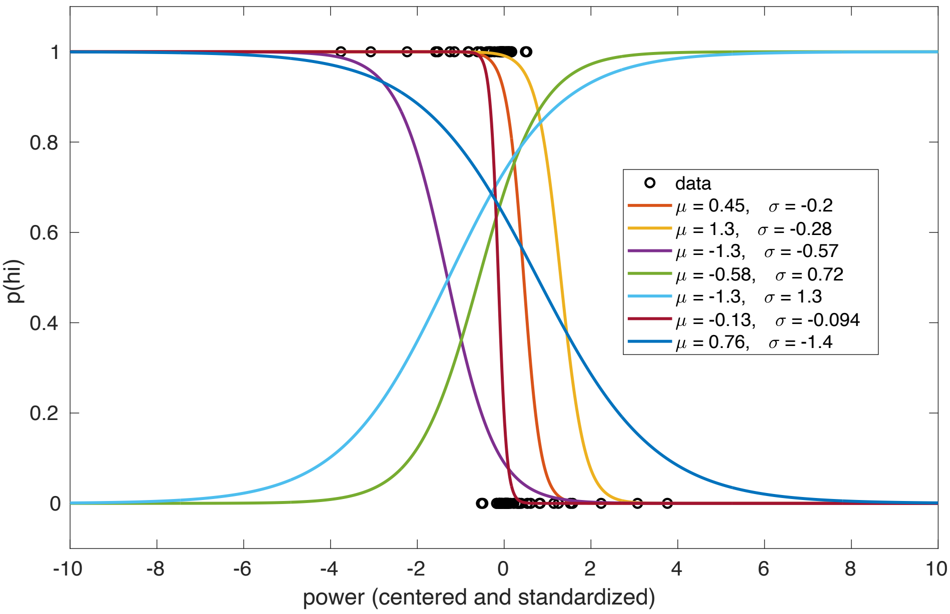


**Figure S8. Graphs of logistic functions for several parameter values drawn from the prior distribution specified above.** To show the prior graphs in relation to the data, by way of example, power values of ROI S1 are depicted as black circles. Specifically, the x- and y-values of the data points correspond to the values in vectors $\hat{\mathbf{P}}$ and $\hat{\mathbf{S}}$, respectively.

According to Bayes' rule, the probability of the data given the model (a.k.a. model evidence) is

$$p_{\text{model}} \quad = \quad \iint p(\hat{\mathbf{S}}, \mathbf{x}|\mu, \sigma)\ \mathcal{N}\left(\begin{bmatrix} \mu \\ \sigma \end{bmatrix};\ \mathbf{0}, \mathbf{I}\right) d\mu\, d\sigma,$$

where for the power and connectivity models, $\mathbf{x}$ is substituted by $\hat{\mathbf{P}}$ and $\hat{\mathbf{C}}$, respectively. In our implementation, we compute this integral using standard Monte Carlo integration with $10^5$ samples.

The described procedure results in $N_{\text{pow}} = 6$ power and $N_{\text{conn}} = 15$ connectivity values per frequency band. The model evidence is computed for all individual power and

connectivity values at all $N_{\text{freq}} = 3$ frequency bands. The resulting model evidence values are denoted by $p_{\text{pow}}^{ml}$ and $p_{\text{conn}}^{nl}$ with indices $m$, $n$, and $l$ coding for the different power, connectivity and frequency values, respectively. The Bayes factors reported in the manuscript represent the ratio of averaged model evidences:

$$
\begin{aligned}
BF_{\text{pow/conn}} &= \frac{N_{\text{conn}} \sum_{m=1}^{N_{\text{pow}}} \sum_{l=1}^{N_{\text{freq}}} p_{\text{pow}}^{ml}}{N_{\text{pow}} \sum_{n=1}^{N_{\text{conn}}} \sum_{l=1}^{N_{\text{freq}}} p_{\text{conn}}^{ml}} \\
BF_{\text{conn/pow}} &= \left(BF_{\text{pow/conn}}\right)^{-1}
\end{aligned}
$$
